# Multi‐Site Hymenoptera Stings Mark a High‐Risk Subgroup in the Emergency Department

**DOI:** 10.1002/kjm2.70220

**Published:** 2026-05-07

**Authors:** Shih‐Fen Tseng, Shum‐Shin Lin, Che‐Cheng Su, Yu‐Jang Su

**Affiliations:** ^1^ Department of Emergency Medicine Taoyuan General Hospital, Taoyuan, Ministry of Health and Welfare Taoyuan Taiwan; ^2^ Institute of Health Policy and Management, College of Public Health, National Taiwan University Taipei Taiwan; ^3^ Toxicology Division, Emergency Department MacKay Memorial Hospital Taipei Taiwan; ^4^ Department of Emergency Medicine Mackay Memorial Hospital Taipei Taiwan; ^5^ Department of Nursing Yuanpei University of Medical Technology Hsinchu Taiwan; ^6^ Department of Medicine MacKay Medical College New Taipei City Taiwan; ^7^ MacKay Junior College of Medicine Nursing and Management Taipei Taiwan

To the Editor,

1

Hymenoptera stings are a common cause of emergency department (ED) visits, with presentations ranging from mild local reactions to systemic toxicity. Clinical analyses of Hymenoptera stings in northern Taiwan have identified advanced age (≥ 65 years), male sex, and multiple sting sites as primary risk factors for severe systemic reactions and hospitalization [1, 2]. While multiple stings are a recognized risk factor for severe outcomes, the clinical significance of anatomical distribution remains less well defined. Here, we present a brief analysis highlighting multi‐site stings as a practical marker of increased clinical burden.

We conducted a retrospective study of patients presenting with Hymenoptera envenomation at two hospitals in northern Taiwan between April 2021 and March 2023. Collected data included demographics, number of stings, anatomical distribution (head/neck, trunk, limbs, or multi‐site), and ED length of stay (LOS).

A total of 266 patients were included (mean age, 46.8 years; 58.6% male). Most stings involved the limbs (69.5%), followed by the head/neck (16.9%), multi‐site involvement (9.1%), and the trunk (4.5%). The number of stings ranged from 1 to 10 and showed a weak but statistically significant correlation with ED LOS (*R* ^2^ = 0.025, *p* < 0.001).

Patients with multi‐site stings had substantially longer ED stays (146.6 ± 185.4 min) compared with those with single‐site stings (58.5–65.9 min, *p* < 0.001) (Figure 1). Patients in the multi‐site group were also significantly older than those in the single‐site groups (56.2 ± 14.7 vs. 43–47 years, *p* = 0.012) [3]. Notably, hospital admission occurred only in the multi‐site group (4.2%), whereas all patients with single‐site stings were discharged. Although severe complications were rare and no deaths occurred, multi‐site involvement consistently corresponded to a higher sting burden and greater clinical resource utilization.

**FIGURE 1:**
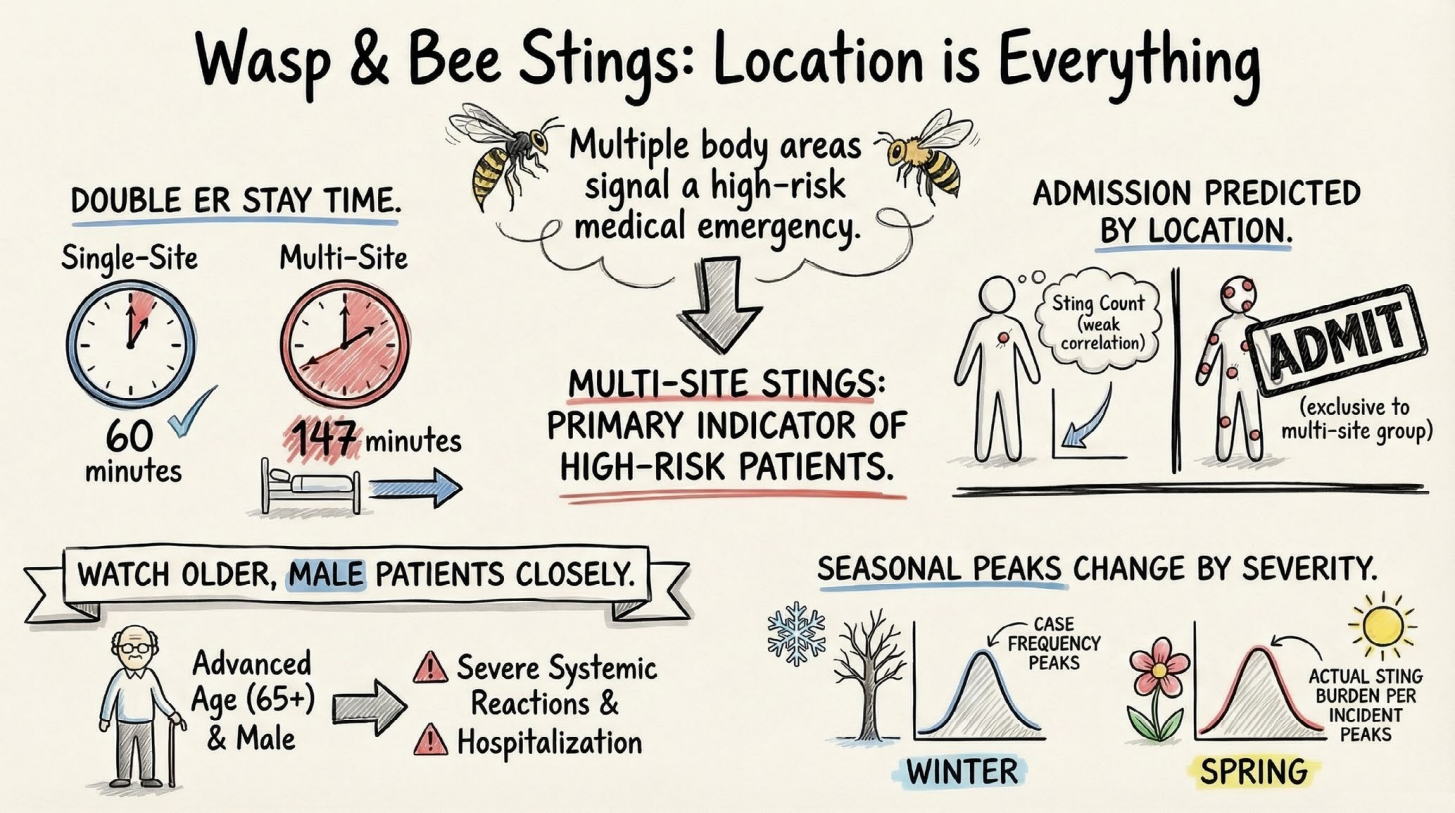
Multi‐site stings are the primary indicator of high‐risk patients, reflecting a greater systemic venom burden compared to single‐site stings. Patients with multi‐site involvement demonstrate significantly prolonged ED stay times (147 vs. 60 min) and are more likely to require hospital admission, whereas sting count alone shows weak correlation with outcomes. Advanced age (≥ 65 years) and male sex are associated with increased risk of severe systemic reactions and hospitalization, warranting closer monitoring. Seasonal variation differs by severity: Overall case frequency peaks in winter, while the actual burden of stings per incident is higher in spring. These findings highlight that sting distribution, rather than count alone, is critical for risk stratification and clinical decision‐making.

These findings suggest that multi‐site envenomation may serve as a simple and clinically applicable indicator of higher risk exposure. Unlike sting count alone—which demonstrated only a weak correlation with LOS—multi‐site distribution likely reflects both increased venom load and prolonged exposure during swarm attacks.

This study has several limitations, including its retrospective design and restriction to two centers, which may limit generalizability. Additionally, detailed immunologic data and long‐term outcomes were not available.

In conclusion, in northern Taiwan, Hymenoptera envenomation predominantly affects the extremities and occurs year‐round, with case frequency peaking in winter and per‐incident sting burden peaking in spring. Multi‐site envenomation identifies a high‐risk subgroup—typically older patients who sustain more stings and are at increased risk of prolonged ED stays and systemic complications—warranting heightened clinical vigilance and targeted preventive strategies.

## Conflicts of Interest

The authors declare no conflicts of interest.

## Data Availability

The data that support the findings of this study are available on request from the corresponding author. The data are not publicly available due to privacy or ethical restrictions.
